# Supplementary material for: KIFC1 Is Associated with Basal Type, Cisplatin Resistance, PD-L1 Expression and Poor Prognosis in Bladder Cancer
Source: J Clin Med. 2021 Oct 21;10(21):4837. doi: 10.3390/jcm10214837 (PMC8584707; doi:10.3390/jcm10214837)
Supplement: Supplementary file 1 [file jcm-10-04837-s001.zip › jcm-1399035-supplementary.pdf]

Supplementary Table S1. Clinicopathologic characteristics of 78 bladder cancer patients who were treated with cystectomy from Hiroshima cohort.

Number of cases	78
Median age (years)	68 (46-85)
Gender	
Female	
Male	
Median follow-up periods (months)	34 (1-158)
Histological grade	
Low	15
High	63
Pathological T stage	
pTis	8 (10%)
pT1	20 (26%)
pT2	26 (33%)
pT3	23 (29%)
pT4	1 (1%)
Venous invasion	
Negative	62 (79%)
Positive	16 (21%)
Lymphatic invasion	
Negative	46 (59%)
Positive	32 (41%)
Pathological N stage	
Negative	66 (85%)
Positive	12 (15%)

Supplementary Table S2. Clinicopathologic characteristics of 50 bladder cancer patients who were treated with cystectomy from Kure cohort.

Number of cases	50
Median age (years)	73 (53-86)
Gender	
Female	17
Male	33
Median follow-up periods (months)	26 (1-148)
Histological grade	
Low	0
High	50
Pathological T stage	
pTis	3 (6%)
pT1	3 (6%)
pT2	11 (22%)
pT3	23 (46%)
pT4	10 (20%)
Venous invasion	
Negative	34 (68%)
Positive	16 (32%)
Lymphatic invasion	
Negative	29 (48%)
Positive	21 (42%)
Pathological N stage	
Negative	33 (66%)
Positive	17 (34%)

Supplementary Table S3. Clinicopathologic characteristics of bladder cancer patients from GSE13507, GSE32548, and GSE48277.

GSE13507	
Number of cases	165
T stage	
Ta	24
T1	80
T2	31
T3	19
T4	11
GSE32548	
Number of cases	130
T stage	
Ta	40
T1	51
≥T2	38
Tx	1
GSE48277	
Number of cases	72
T stage	
T2	42
T3	22
T4	8

Supplementary Table S4. Proliferation signature.

Gene	ANLN, ASF1B, ASPM, ATAD2, AURKA, AURKB, BUB1, BUB1B, C12orf48, C15orf23, C16orf59, C17orf53, C1orf112, CCNA2, CCNB1, CCNB2, CCNE2, CDC20, CDC25A, CDC25C, CDC45, CDCA2, CDCA3, CDCA5, CDCA8, CDK1, CDKN3, CENPA, CENPE, CENPF, CENPL, CENPN, CEP55, CHAF1B, CHEK1, CKAP2L, CKS1B, DEPDC1, DEPDC1B, DLGAP5, DONSON, E2F2, EXO1, FAM64A, FAM83D, GINS2, HJURP, HMGB2, HMMR, KIAA1524, KIF11, KIF14, KIF18A, KIF20A, KIF20B, KIF23, KIF2C, KIF4A, MCM2, MCM7, MELK, NCAPG, NUF2, NUSAP1, OIP5, PKMYT1, PLK4, POLA2, POLE2, POLQ, PRC1, PRR11, RACGAP1, RAD51AP1, RFC3, RRM2, SKA3, TK1, TOP2A, TRIP13, TTK, TYMS, UBE2T, UHRF1, ZWINT
